# The influence of the gut microbiota on B cells in autoimmune diseases

**DOI:** 10.1186/s10020-025-01195-5

**Published:** 2025-04-22

**Authors:** Lun He, Xin Li, Shan Jiang, Yanhua Ou, Shanshan Wang, Na Shi, Zhongshan Yang, Jia-li Yuan, Gregg Silverman, Haitao Niu

**Affiliations:** 1 Key Laboratory of Viral Pathogenesis & Infection Prevention and Control (Jinan University), Ministry of Education; Guangzhou Key Laboratory for Germ-free Animals and Microbiota Application, School of Medicine, Jinan University, Guangzhou, 510632 China; 2 Yunnan Provincial Key Laboratory of Molecular Biology for Sinomedicine, Yunnan University of Chinese Medicine, Kunming, Yunnan, 650500 China; 3 Division of Rheumatology, New York University School of Medicine, New York, NY 10016 USA

**Keywords:** Gut microbiota, B lymphocytes, Autoantibodies, IgA, Bregs, Germinal centers, Autoimmune diseases

## Abstract

Mounting evidence shows that gut microbiota communities and the human immune system coexist and influence each other, and there are a number of reports of a correlation between specific changes in gut microbiota and the occurrence of autoimmune diseases. B lymphocytes play a central role in the regulation of both gut microbiota communities and in autoimmune diseases. Here, we summarize evidence of the influence of gut microbiota-B cell pathways on autoimmune diseases and how B cells regulate microorganisms, which provides mechanistic insights with relevance for identification of potential therapeutic targets and related fields.

## Introduction

Our microbiome is an important part of the human body, with especially large communities in the gastrointestinal tract, reproductive tract, urinary tract, skin, oral cavity, respiratory tract, and other locations (Aggarwal et al. 2023). The number of microbial cells in the gut microbiota in humans is estimated to exceed 100 trillion cells, and there are many types of organisms, including bacteria, fungi, and viruses, among which bacteria are the predominant residents (Gill et al. 2006) which participate in human physiological activities and affect fundamental mechanisms that maintain homeostasis in the human body. When there is a serious imbalance within the human gut microbiota community, the protective effect of gut microbiota on the human body can weaken, conveying vulnerability to a variety of diseases (Guo et al. 2022; Bajaj 2019; Meng et al. 2018). The immune system’s main function is to prevent infection and eliminate invasive pathogens while maintaining tolerance for self-antigens in health and preserving tissue organization, which plays a vital role in maintaining homeostasis and is required for health (Reider et al. 2020). When the mechanism of maintaining autoimmune tolerance fails, the immune system can mistakenly attack and destroy healthy cells and tissues, resulting in an autoimmune disease (Casella et al. 2020; Trier and Houen 2023). However, in the study of the relationship between gut microbiota and autoimmune diseases, the effect of gut microbiota on B cells has been understudied, which offers new research directions for exploration of the mechanisms responsible for triggering and subsequent development of autoimmune diseases, and which may also suggest new treatment strategies.

## Gut microbiota, immune system, and autoimmune diseases

### Association of the gut microbiota with the immune system

A considerable part of the human immune system is centered in the intestine, with a large number of immune cells in the body, such as natural killer(NK)cells, T cells, and B cells, concentrated in the intestinal mucosa, playing critical immune functions (Shao et al. 2023). The gut microbiota and the immune system have very close reciprocal connections (Bhatt et al. 2017). The intestinal flora influences the immune system to function. The signals arising from the changes in the gut microbiota can be transmitted from the intestine to other parts of the body through the immune system. This process is called an “axis,” which causes the body to produce a corresponding response (Ost and Round 2018; Cryan et al. 2019). Since the human body’s adaptive immune system has the characteristics of molecular specificity, the memory of past exposure, and self-tolerance, there is the capacity to effectively respond to the various shifts within and responses from gut microbiota, which promote the symbiotic relationship between the host and the gut microbiota, and then once again reinforce host immune defenses to maintain health (Belkaid and Harrison 2017). However, the composition and abundance, as well as the function of the gut microbiota may become excessively altered, and such changes can alter the balance of the gut microbiota, which is commonly referred to as gut microbiota dysbiosis (Chen et al. 2021). This dysbiosis may contribute to the development of a range of diseases, such as colorectal cancer and other gut-localized disorders, and it has been demonstrated that the gut microbiota is capable of releasing a wide range of metabolites, proteins and macromolecules that may influence the development and progression of colorectal cancer (El Tekle et al. 2024). However, interventions with probiotics and prebiotics can modify the gut microbiota to favor the immune response and support immunotherapy (Bevilacqua et al. 2024; Jiang and Zhang 2024).

### Mechanisms affecting gut microbiota dysbiosis and the development of autoimmune diseases: the role of the molecular mimicry hypothesis

Gut microbiota dysbiosis has also been observed in systemic diseases, including autoimmune diseases (Belvoncikova et al. 2022). The association between autoimmune diseases and microbiota has become a research hotspot in exploring the relationship between gut microbiota and diseases. However, there is currently an inadequate understanding of the mechanisms by which shifts in the gut microbiota may contribute to the development of autoimmune diseases. Still, a range of evolving hypotheses has been proposed. One of the key hypotheses is known as molecular mimicry, a gradually evolving theory that was initially based on the idea that certain microorganisms and human cells or cellular elements have the same or similar antigenic epitopes. Following an infection within the human body, an immune response is induced against microbial antigens, which can erroneously activate a similar set of human cells and extracellular components, ultimately contributing to the development of various autoimmune diseases (Rojas et al. 2018). Classic examples are streptococcal antigens and rheumatic fever, or post-streptococcal glomerulonephritis, whereas Epstein-Barr virus (EBV) encoded protein, latent membrane protein 1 (LMP1) and the human protein, myelin basic protein (MBP), have high homology, and this mimicry has been postulated to the basis by which this viral infection can contribute to multiple sclerosis (Gabibov et al. 2011; Sospedra and Martin 2016). Similarly, Coxsackie virus infection has been proposed to trigger an immune response that attacks the β cells of the islets, which leads to type I diabetes mellitus (Dotta et al. 2007). The key to molecular mimicry is the similarity between exogenous peptides and self-peptides, which favors the activation of autoreactive T or B cells (Rojas et al. 2018). This hypothesis suggests that the presence of bacterial antigens similar to self-antigens during changes in the intestinal microbiota may trigger molecular mimicry responses, which in turn contribute to the onset and progression of autoimmune diseases, such as spondyloarthropathies (SpA). Notably, the presence of the human leukocyte antigen (HLA) class I, B-27 (HLA-B27) is strongly correlated with SpA; there is a strong correlation between autoantigenic peptides derived from HLA-B27 (LRRYLENGK) and peptides derived from intestinal microorganisms (Escherichia coli, Klebsiella nitrogenase, Pseudomonas aeruginosa, Salmonella typhimurium, and Bacillus megaterium) with similar sequences, which may activate an immune response against autoantigens (Fantini et al. 2009; Tie et al. 2023). In addition, collagen type 2 (Coll 261–273) in rheumatoid arthritis was found to share sequence similarity with Haemophilus parasuis, which may drive inflammatory responses against self-antigens and enhance IL-17 production, thereby contributing to the onset and progression of rheumatoid arthritis (Di Sante et al. 2021). Host immune system responses to gut microbes and their metabolites also influence molecular mimicry processes, and overactivation or defects in immune regulation or tolerance may lead the immune system to mistakenly attack its own tissues, increasing the risk of autoimmune disease (Rojas et al. 2018; Felix et al. 2018). Although this mechanism is not well understood, the molecular mimicry theory has become one of the most important frameworks for studying the relationship between gut microbes and autoimmune diseases, providing useful insights for future studies.

### Dysbiosis of the gut microbiota and autoimmune diseases

With the intensive study of gut microbiota, there is more and more evidence that abnormalities and imbalances of gut microbiota are connected to the pathogenesis of some autoimmune diseases, most of which are characterized by a marked imbalance in the abundance and diversity of the intestinal microbiota relative to that of healthy populations (Table 1). It has been postulated that certain interventions, such as diet intervention (Vieira et al. 2014), fecal microbiota transplant (FMT) (Weingarden and Vaughn 2017) and probiotic therapy (de Oliveira et al. 2017), etc., may be used to improve the intestinal micro-ecological environment, to achieve the prevention and/or control of autoimmune diseases, and such treatment methods appear to have great potential.

**Table 1:** Research on the association between gut microbiota dysbiosis and autoimmune disease

Autoimmune disease	Description of Dysbiosis
Type 1 Diabetes (T1D)	Dysbiosis in the gut microbiota is associated with T1D onset. Patients exhibit an increased abundance of genera like *Bacteroides* and *Clostridium*, and reduced levels of *Lactobacillus* and *Bifidobacterium* (Han et al. 2018; Del Chierico et al. 2022).
Rheumatoid Arthritis (RA)	Alterations in gut microbiota composition are observed in RA patients, with increased levels of *Lactobacillus Bifidus* and *Prevotella* (Maeda and Takeda 2019; Zhao et al. 2022).
Systemic Lupus Erythematosus (SLE)	Dysbiosis in SLE patients includes decreased levels of *Firmicutes* and increased levels of *Bacteroidetes* (Mu et al. 2015; Ma et al. 2019).
Inflammatory Bowel Disease (IBD)	IBD patients demonstrate significant differences in gut microbiota compared to healthy individuals, with reductions in *Faecalibacterium* and increases in *Escherichia coli* (Ni et al. 2017; Haneishi et al. 2023).
Autoimmune Hepatitis (AIH)	Dysbiosis in gut microbiota may be associated with AIH, and increases in *Veillonella*, and decreases in *Bifidobacterium* are associated with disease states (Wei et al. 2020; Cheng et al. 2022).
Autoimmune Thyroid Disease	Dysbiosis in the gut microbiota is implicated in autoimmune thyroid diseases. Studies have found that patients with hypothyroidism have a decrease in *Firmicutes*, and patients with hyperthyroidism tend to have a decrease in *Bifidobacteria* and *Lactobacilli* (Virili et al. 2018; Jiang et al. 2022).
Psoriasis	Patients with psoriasis exhibit dysbiosis of the gut microbiota characterized by an increase in *Escherichia coli* and *Ruminococcus* and a decrease in *Lachnospira*, *Faecalibacterium* and *Akkermansia muciniphila* (Buhaș et al. 2022).

## B cells in autoimmune diseases

B lymphocytes, also simply known as B cells, arise in the bone marrow from pluripotent stem cells. B cells can differentiate into a variety of cellular subsets, including B-1 cells, B-2 cells, marginal zone (MZ) B cells, lymphocyte-like cells, regulatory B (Breg) cells, plasma cells, memory B cells, Aging-associated B cells (ABC) and others (Allman and Pillai 2008). B cells can play a variety of biological functions. On the one hand, they differentiate into plasma cells to produce antibodies, which play an important role in humoral immunity (LeBien and Tedder 2008), and they also play non-immunoglobulin roles in uptake, processing, and presentation of protein antigens as antigen-presenting cells (APCs). When stimulated they can also secrete a variety of cytokines, including TNF-a, IL-10 and IL-6 (Rastogi et al. 2022; Glass et al. 2022).

### Autoantibodies in autoimmune diseases

One of the hallmarks of many autoimmune diseases is the presence of autoantibodies, which are now widely used in clinical diagnosis. Some autoantibodies have also been shown to be directly involved in the pathogenesis of specific autoimmune diseases (Ludwig et al. 2017). Autoantibodies can promote tissue damage through antibody-dependent cytotoxicity (ADCC). The mechanism is through the interaction of autoantibody-bound target tissue antigens with Fc receptors (FcRs) of macrophages, neutrophils, NK cells, and other effector cell types. For example, mice susceptible to lupus with mutations in the IgG FcR gene exhibit reduced participation of effector cells, which alleviates glomerulonephritis and improves renal output, indicating that IgG FcR mutations have a protective role in this mouse model of lupus (Tipton et al. 2018).

Antiphospholipid autoantibodies have been found to modulate B-cell function and contribute to the pathogenesis of autoimmune diseases particularly in lupus-prone mouse models. In SLE, antiphosphatidylserine antibodies produced by B1 cells contribute to the development of lupus nephritis by activating the TLR/Syk pathway, as demonstrated in MRL/Lpr model (Ma et al. 2023). In RA, disease progression has been associated with B-cell activation and the presence of anti-citrullinated protein antibody (ACPA) antibodies (Qin et al. 2023). Specifically, a hallmark of the disease is the presence of anti-modifier protein antibodies (AMPA), which are autoantibodies that target antigens with different post-translational modifications, including anti-citrullinated protein antibodies (ACPA), anti-carbamoylated protein antibodies (anti-CarP) and anti-acetylated protein antibodies (AAPA). The targeted antigens are highly cross-reactive with AMPA, which activates B cells in the body and promotes disease progression (Kissel et al. 2020). It was found that in the K/BxN mouse model, B-cell activation and autoantibody production may be increased in the context of an intensified Tfh cell response due to P2RX7 gene deletion, thereby exacerbating the development of autoimmune arthritis (Felix et al. 2019).

In addition, immune complexes can be formed or deposited in tissues by activating complement, inducing tissue damage through complement-dependent cytotoxicity (CDC). In one study in a mouse model of lupus nephritis, blocking B cell costimulation, combined with cyclophosphamide treatment, reduced immune complex deposition and glomerulonephritis and preserved kidney function (Yu et al. 2017). More recently, treatment with an anti-CD20 antibody that depletes B cells was also shown to greatly ameliorate early disease in both mouse models and human studies (Gong et al. 2016; Hartinger et al. 2022; van Schaik et al. 2022). However, other studies have shown that antibodies can also play an important anti-inflammatory effect, thereby limiting or even inhibiting the occurrence of autoimmune diseases (Dalakas 2022). Whether an antibody promotes or inhibits inflammation is related to the specificity, type, and concentration of the antibody. Non-IgG antibody isotypes, such as IgM, can have different effects (Strait et al. 2015). Some subclasses of IgG molecules express pro-inflammatory or even anti-inflammatory activity, depending on the specific glycosylation pattern (Nimmerjahn and Ravetch 2008). For example, galactosylated IgG1 links FcγRIIB and Dectin-1 to block complement-mediated inflammation (Karsten et al. 2012). Importantly, antibody glycosylation can also be regulated during the immune response (Nimmerjahn and Ravetch 2008).

### IgA: multifaceted roles in immunity and autoimmunity

In the process of antibody response, antigen encounters with the membrane-associated antigen receptor activate B cells, and the expressed Ig isotype on the cell membrane becomes secreted. Commonly, after cellular activation, the IgM is generally expressed on resting B cells, which can lead to the secretion of other isotypes such as IgG, IgA, IgE, etc. These play important specialized roles in the body. Herein we focus on IgA, which is the predominant product of mucosal tissues and is the most highly produced isotype in the human body (Wines and Hogarth 2006). In the serum of a healthy individual, the concentration of immunoglobulin A (IgA) is second only to that of IgG among immunoglobulins, and these glycoproteins are usually in monomeric form (mIgA), while secretory IgA (SIgA), which is released directly onto mucosal surfaces of the gastrointestinal tract, genitourinary tract and respiratory tract, is predominantly a dimeric molecule incorporating the accessory J chain. Through its constant regions, IgA can bind and interact with specific types of receptors on a variety of cells. Among them, the best known is the IgA receptor, FcαRI, which is a member of the Fc receptor immunoglobulin superfamily and is expressed by many bone marrow cells (Aleyd et al. 2015).

Studies have confirmed that monomeric IgA binds to FcαRI with low affinity and activates immunoreceptor tyrosine-based activation motif (ITAM) signaling, leading to partial phosphorylation of FcRγ-ITAM complex, thereby mediating inhibitory signals (van Gool and van Egmond 2020). Monomeric IgA is believed to have important anti-inflammatory effects (Mkaddem et al. 2014), while IgA immune complexes, such as those with pathogen antigens complexed with IgA antibodies, bind to FcαRI, which results in cross-linking of these cell-membrane associated receptors to induce pro-inflammatory responses (Bakema and van Egmond 2011). It has been found that the IgA immune complex-FcαRI interaction may trigger inflammatory reactions by neutrophils and macrophages in the joints of patients with RA, and it has been shown that blocking the interaction between FcαRI and IgA RF in macrophages can lead to a local decrease in the level of the proinflammatory cytokine TNF-α (Anquetil et al. 2015). Therefore, IgA immune complexes may contribute to the pathogenesis of autoimmune diseases. However, as IgA antibodies secreted onto mucosal surfaces can aid clearance of pathogenic microbes, IgA deficiency may increase susceptibility to infection. There is also evidence that individuals with IgA deficiency have a higher incidence of autoimmune diseases (Singh et al. 2014). Cumulatively, these studies have shown that IgA plays an important role in maintaining the balance of mucosal immunity and eliminating pathogens, but that the lack of IgA, or excessive IgA immune complexes can also be harmful. To restore balance, we can propose some treatment approaches. On the one hand, we could seek to prevent infection by vaccination aimed at stimulating IgA responses. In theory, through immune responses that recruit functions mediated by FcαRI, we may also reduce inflammation by inducing the inhibitory signal of ITAM by FcαRI, or the blockade of pro-inflammatory stimulation of FcαRI with monoclonal antibodies or peptides may reduce IgA-induced inflammation and thereby prevent tissue damage (Breedveld and van Egmond 2019).

### Cytokine involvement in autoimmune diseases

In addition to the production of antibodies, B cells can also influence immune responses through the local secretion of immunomodulatory cytokines, including pro-inflammatory cytokines such as IFN-γ, IL-6, and TNF-α (Zhang and An 2007), or by the secretion of anti-inflammatory cytokines IL-10, TGF-β, and IL-35 (Dang et al. 2014; Shen et al. 2014).

Recently, it was found that IL-35^+^ B cells subdue NK cell responses and exert anti-tumor effects (Li et al. 2022). In many patients, IFN-γ plays central roles related to the pathogenesis of SLE (Liu et al. 2022). In the MRL/lpr mouse model, IFN-γ can induce the formation of germinal centers that foster autoimmune responses through the transcription factor BCL-6 induced in the B cells (Jackson et al. 2016). Moreover, in the MRL/lpr model, mice with genetic deficiency for IFN-γ receptors display greatly attenuated severity of the autoimmune disease (Hron and Peng 2004). IL-6 can promote the activation and proliferation of B cells and also influences the differentiation of Th17 cells, which are thought to drive disease. IL-6 also plays a multitude of important roles in chronic inflammation (Kishimoto 2019). For example, in rheumatoid arthritis (RA), a number of studies have reported an increase in serum IL-6 levels in clinically active RA patients (Srirangan and Choy 2010), which can be significantly decreased by effective therapeutic intervention (Madhok et al. 1993), suggesting that IL-6 that is produced in the RA-affected joint may be a primary driver of the progression of RA disease. Therefore, we may prevent the loss of immune tolerance of B and T cells through targeted B cell therapy by administration of anti-CD20 antibodies (such as rituximab) (Min et al. 2022) and reduce the downstream inflammatory response that further promotes the spreading of pathogenic autoimmunity.

### The regulatory role of Bregs in autoimmune diseases

Regulatory B cells (Bregs), an important subset of functional B cells capable of producing anti-inflammatory cytokines, have been known to play an important regulatory role in maintaining tolerance and inhibiting inflammatory autoimmune responses (Mauri and Bosma 2012; Tan et al. 2022). The ontogeny of Bregs varies, as they may originate from different B cell subsets. Their production of IL-10, TGF-β, and IL-35 inhibits the proliferation, differentiation, and related biological activities of inflammatory T cells and other pro-inflammatory lymphocytes, thereby preventing the occurrence of pathological immune response (Rosser and Mauri 2015).

It has been fully proved by animal models and clinical studies that the number and functional activity of Bregs are usually negatively correlated with the development and severity of autoimmunity, and injury to Bregs can promote the development of autoimmunity (Miyagaki et al. 2015). For many years, it has been believed that development of Bregs are primarily regulated by the production of IL-10 and are known as potent inhibitors of autoimmune inflammation (Mauri and Bosma 2012). It has been demonstrated in mouse autoimmune models of SLE, RA, and MS that the Breg cells producing IL-10 have an important protective effect on the development of autoimmunity (Moudgil and Choubey 2011). This suggests, for the treatment of autoimmune disease, in addition to using conventional immune inhibitors, we can also pursue targeted modification of Bregs as a treatment strategy, which could theoretically increase the activity of Bregs and/or the number of amplifying Bregs (Mauri and Menon 2017). The concept presents multiple open questions and problems, such as maintenance of normal activity of amplified Bregs, and lack of understanding of what level of increase may be appropriate, as too little may not be able to achieve proper treatment effect, while too much may increase the risk of immune disease. In spite of these challenges, this still shows a broad potential for the treatment of autoimmune and even other inflammatory diseases. It is believed that through further studies, this approach will be evaluated for the treatment of immune diseases in the future.

### Association of dysregulated B-cell immune tolerance with autoimmune diseases

The link between B-cell immune tolerance and autoimmune diseases has been a research topic of great interest in the field of immunology. Recent studies have delved into the close connection between dysregulation of B-cell immune tolerance and the pathogenesis of autoimmune diseases from several aspects. First, aberrant activation of B cells and escape from autoimmune tolerance checkpoints is one of the key mechanisms in the pathogenesis of autoimmune diseases (Bonasia et al. 2021; Corneth et al. 2022). B-cell tolerance checkpoints are an important immunoregulatory mechanism aimed at preventing B cells from generating an aggressive immune response to self-antigens. These checkpoints, which involve central and peripheral tolerance, modulate B-cell activation and function through negative selection and immunosuppressive factors. Dysregulated tolerance checkpoints may contribute to the development of autoimmune diseases (Wu et al. 2021). Researchers have found that most B cells produced in the bone marrow are autoreactive to some degree, but in healthy individuals, they become unresponsive through a process of negative selection. However, early B-cell tolerance checkpoints in patients with autoimmune diseases are often defective, leading to an accumulation of self-reactive B cells that can promote autoimmunity by presenting self-antigens to T cells (Nemazee 2017). Studies of patients with autoimmune diseases, such as rheumatoid arthritis (RA) and systemic lupus erythematosus (SLE), have revealed the presence of large amounts of autoantibodies in these patients, which are usually produced by self-antigen-specific B cells, and such abnormally activated B cells may escape the regulation of immune tolerance checkpoints, leading to the production of pathogenic autoantibodies, which in turn can trigger inflammatory responses and tissue damage (Bonasia et al. 2021; Wang et al. 2015). In addition, therapeutic strategies targeting dysregulation of B-cell immune tolerance have become a hot topic of current research. Several studies have found that the pathogenesis and progression of autoimmune diseases can be significantly influenced by regulating the function of B-cell immune tolerance checkpoints. One study found that the induction of tolerance to autoantigens may have a therapeutic effect in the K/BxN mouse, and autoimmune rheumatoid arthritis could be inhibited by inducing B cells to be tolerant to autoantigens through a hybrid nanoparticle (Brzezicka et al. 2022). PI3Kδ is an isoform of phosphatidylinositol 3-kinase (PI3K), which plays a major regulatory role in B cells (Okkenhaug 2013). It has been found that when PI3Kδ is over-activated, it leads to aberrant activation and dysfunction of B cells, which breaks down multiple immune tolerance checkpoints both centrally and peripherally, and targeting the regulation of the PI3Kδ signaling pathway could be one of the potential strategies for the treatment of autoimmune diseases (Lau et al. 2020). EZH2 is a histone methyltransferase, which regulates chromatin structure and gene expression through methylation modification. In systemic lupus erythematosus, the immune tolerance of B cells is disrupted, leading to overproduction of autoantibodies and exacerbation of autoimmune responses, and inhibition of EZH2 repairs this immune tolerance and reduces autoantibody production in pristane-induced lupus mice (Yang et al. 2023). Overall, there is a close association between dysregulation of B-cell immune tolerance and autoimmune diseases, and an in-depth study of this association may help us better understand the pathogenesis of autoimmune diseases and provide new targets and strategies for their treatment.

## Interactions between gut microbiota and B cells: does this affect the occurrence and development of autoimmune diseases?

### Gut microbiota regulate B cells and autoantibodies

The gut microbiota plays an important role in maintaining tolerance to autoimmunity and can contribute to the development and persistence of autoimmunity (Botía-Sánchez et al. 2021). Microbial antigens can directly activate the differentiation of B cells into plasma cells and influence the formation of the B cell repertoires, a process that is significantly influenced by and dependent on the gut microbiota (Chen et al. 2022). Previous studies have found that NLRP12 modulates inflammation at the molecular level, exhibiting anti-inflammatory effects that help maintain a healthy gut microbiota, thereby preventing the onset of colitis (Chen et al. 2017). However, in Nlrp12-deficient MRL/lpr mice, it was found that Nlrp12 deficiency significantly altered the gut microbiota of male mice, inhibited auto-reactive B-cell responses, and reduced production and renal deposition of autoantibody, thereby attenuating the inflammatory response to B6/lpr mice (Abdelhamid et al. 2023). Another study found that the transplantation of feces from a systemic lupus erythematosus (SLE) model, B6.NZM-Sle1^NZM2410/Aeg^Sle2^NZM2410/Aeg^Sle3^NZM2410/Aeg^/LmoJ congenic mouse (TC) into germ-free mice induced anti double-stranded DNA (anti-dsDNA) antibodies and innate immune responses (Ma et al. 2019). Mice with collagen-induced arthritis (CIA) are a common animal model of rheumatoid arthritis, and CIA-susceptible mice with regularized microbiota have higher rates of arthritis than germ-free mice, higher concentrations of IL-17, higher proportions of CD8^+^ T and Th17 cells, and lower proportions of dendritic cells, B-cells, and Tregs, suggesting that the gut microbiota influences arthritis susceptibility (Liu et al. 2016).

Graves’ disease (GD) is a common systemic autoimmune disorder mediated by autoantibodies to the thyrotropin hormone receptor, and studies have suggested that the underlying pathogenesis of GD is caused by dysregulation of the gut microbiota, antigen mimicry and Th17/Treg imbalance (Hou et al. 2021). In Hashimoto’s thyroiditis (HT), elevated concentrations of autoantibodies reactive against thyroglobulin are associated with gut microbial dysbiosis (Cayres et al. 2021). Changes in the gut microbiota of CIA mice were found to lead to increased gut barrier permeability and mucosal inflammation, which prompted B-cell activation and production of autoantibodies, while Th17 and B-cells infiltrated the mucosa, triggering a systemic autoimmune response. The microbiota may influence the development of autoimmune arthritis by regulating autoantibody activation and complement via glycosylation (Liu et al. 2016). The gut microbiota can also influence B-cell function and autoantibody production through interaction with dietary components (such as high fat and high salt diets) (Petta et al. 2018). In addition, Short-chain fatty acid (SCFA), a metabolite of gut microbiota, can promote the differentiation of B cells into antibody-producing cells and influence the host antibody response (Kim et al. 2016).

### Interactions between gut microbiota and IgA in autoimmune disease pathogenesis

In the relationship between gut microbiota and B cells, IgA plays a prominent role. The intestinal mucosa is an important line of defense of immune defense, involving constantly induction of IgA that reacts with the symbiotic bacteria and food antigens. IgA can interact with symbiotic bacteria or food antigens to affect the abundance and diversity of gut microbiota, and gut microbiota are able to regulate the abundance of IgA and its specificity (Bunker et al. 2017). Through this kind of interaction, the IgA helps maintain intestinal homeostasis and has a mucosal protective effect against gut microbiota (Upadhyay and Littman 2022).

Recently, IgA autoantibodies from individuals who are at risk for RA were shown to cross-react with gut bacteria in the Lachnospiraceae and Ruminococcaceae families, suggesting a role for the gut microbiota in the development of autoantibodies in RA (Chriswell et al. 2022). Previous studies have shown that the development of intestinal B lymphocytes is affected by microbial colonization, and early B cell development can also occur in the lamina propria (LP) (Wesemann et al. 2013), where IgA^+^ cells are generated in situ by B220^+^IgM^+^ lymphocytes (Fagarasan et al. 2001). Plasma cells located in the lamina propria of the small intestine produce a large proportion of IgA in the absence of inflammation or immune response (Planer et al. 2016). In one study, it was shown that whole-body IgA production in germ-free (GF) mice was even negligible compared to that in mice with a specific pathogen free (SPF) microbiota (Bunker et al. 2017), suggesting that induction of IgA was highly dependent on the presence of bacterial antigens. For example, Clostridium bacteria, many of which produce short-chain fatty acids (SCFA), have been shown to ferment dietary fiber to produce SCFA, such as acetic acid and butyric acid, which can contribute to the induction of intestinal IgA production through G-protein coupled receptor (GPCR) activation (Mora et al. 2006). However, not all bacteria are capable of robustly inducing IgA production, such as those that are abundant in the gut but do not produce SCFA (Martin-Gallausiaux et al. 2021). Moreover, luminal IgA and systemic IgG can target different gut microbiota constituents (Vujkovic-Cvijin et al. 2022). Lactobacillus rhamnoses (LGG) can induce the production of intestinal IgA, with the main mechanism described to involve the protein p40 derived from LGG activating the epidermal growth factor receptor of intestinal epithelial cells, thereby promoting a protective immune response and preventing inflammation and injury (Shen et al. 2018).

Newborns are known to acquire passive immunity to via IgA through breastfeeding, and this is thought to have a protective effect by preventing the transfer of aerobic bacteria from the neonatal gut to the draining lymph nodes, altering the composition of the gut microbiota. SIgA in breast milk can promote long-term intestinal homeostasis, adding to the evidence for the benefits of breastfeeding (Rogier et al. 2014). Recent research has shown that during aging, the gut microbiota can also regulate IgA expression in pituitary cells (Li et al. 2023). It was observed that in the absence of IgA, the composition of intestinal flora can also be altered (Macpherson et al. 2018; Li et al. 2020a). In addition, in recent years, there has been increasing evidence that the gut microbiota can influence the intestinal mucosal immune system, which is closely related to the development of IgA nephropathy (Ren et al. 2023). In summary, evidencesuggest that the gut microbiota and IgA can interact with each other to exert effects that modulate the immune system. The specific mechanisms of their interactions need to be further investigated.

Here, we outline that IgA can mediate anti-inflammatory or pro-inflammatory responses through different mechanisms of action and is involved in the onset and progression of autoimmune diseases. Therefore, we hypothesize that the gut microbiota can act as a “bridge” through IgA to influence autoimmune diseases, which may provide new ideas for understanding pathogenesis of autoimmune diseases and their potential treatment.

### Impact of Bregs interaction with gut microbiota on autoimmune diseases

Regulatory B cells, termed Bregs, are an important cell subtype that may be elicited by exposure to certain species within the gut microbiota, and these can have immunosuppressive effects in a variety of inflammation and disease models, including models of autoimmune diseases (Botía-Sánchez et al. 2021).

The induction of Bregs may also depend on the presence of bacterial antigens. Studies have found that colonizing germ-free (GF) mice with feces from specific pathogen-free (SPF) mice increases the number of B cells and IL-10 producing cells in the colon tissue of GF mice (Mishima et al. 2019). Toll-like receptors (TLR) are broadly expressed types of innate immune receptors, which can recognize pathogen-related pattern molecules. Different components of Gram-positive and Gram-negative bacteria can stimulate TLR2 and TLR4, respectively. For example, lipopolysaccharide (LPS) is a gram-negative cell wall component that can be recognized by TLR4, and LPS stimulation is mediated by TLR4 signaling, which may contribute to SLE pathogenesis (Mu et al. 2015). A study showed that commensal bacteria in the intestine could interact with TLRs to induce Bregs to produce IL-10 (Luu et al. 2024). Among them, the monopolization of E. coli will promote the production of IL-10 by B10 cells, and it is believed that Bregs rely on bacteria to activate TLR2/TLR4, resulting in an immunosuppressive signaling effect (Maerz et al. 2019). It has been shown that variants in the PTPN22 gene (encoding T-cell protein tyrosine phosphatase 22) are associated with the pathogenesis of RA and can alter the composition of the gut microbiota, leading to a decrease in microbial-derived short-chain fatty acids and a reduction in the inhibitory effect of Bregs cells (Berthelot et al. 2022). Correspondingly, gut microbiota-derived short-chain fatty acids (SCFA) regulate B-cell differentiation through FFA2 receptors in CIA mice, increasing the proportion of Bregs and thereby reducing the inflammatory response in this mouse model of rheumatoid arthritis (Yao et al. 2022).

Aryl hydrocarbon receptor (AhR) is a cytoplasmic receptor and transcription regulator which can bind to a variety of ligands and regulate the development and function of a variety of immune cells (Pernomian, Duarte-Silva and de Barros Cardoso 2020). It is an environmental sensor that allows immune cells to adapt to changes in environmental conditions. Studies have shown that AhR activity can play an important role in controlling the severity of immune diseases, especially in mouse models of lupus (Shinde and McGaha 2018). Specifically, gut microbial-derived SCFAs were reduced in RA patients and arthritic mice, whereas SCFA butyrate supplementation reduced the severity of arthritis (Rosser et al. 2020). Gut microbiota-derived metabolites, especially butyl acid, can increase the production of 5-hydroxytryptamine metabolite 5-hydroxy indole-3-acetic acid (5-HIAA), which binds with AhR to exert transcriptional regulation effects in Bregs, thus affecting the function of Bregs and increasing the production of IL-10 (Rosser et al. 2020). Thus, butyrate supplementation may control a molecular program by activating the AhR, supporting Breg function while inhibiting the development of arthritis (Rosser et al. 2020). In a study on human umbilical cord mesenchymal stem cells (HUMSC) treatment in rats with collagen-induced arthritis (CIA), it was found that HUMSC could regulate the interactions between host immunity and gut microbiota through the AhR, affecting B-cell differentiation, increasing the proportion of B-cells in the Peyer’s Patches (PP) and Lamina Propria Lymphocytes (LPL), and partially reversing arthritis-induced immune disorders (Li et al. 2020b).

In general, Bregs have an important correlation with autoimmunity. Normally, functional and active Bregs can inhibit the development of autoimmunity and vice versa. The gut microbiota can induce Bregs to exert its important physiological functions (especially the production of IL-10) through related pathways. The results of the interaction between gut microbiota and Bregs will further affect the occurrence and development of autoimmune diseases, which suggests that we can intervene and treat autoimmunity through gut microbiota. However, these are all circumstantial links, and the exact mechanisms by which they may occur need to be explored.

### Association of gut microbes with germinal center reactions and autoimmune diseases

Germinal centers (GCs) are specialized microstructures that form within the B-cell follicles of secondary lymphoid organs (Victora and Nussenzweig 2022). GCs usually persist for only a few weeks to months in tissues such as the spleen and lymph nodes and disappear when the source of the infection is cleared. In contrast, chronic GCs are present in tissues such as the intestinal Peyer’s Patch, where B cells also undergo affinity-based selection due to continuous exposure to the intestinal microbiota (Young and Brink 2021; Chen et al. 2020). The germinal centers play multiple roles in the immune response, the most important of which are the promotion of antibody affinity maturation and antibody diversity. In the germinal centers, B cells undergo somatic hypermutation (SHM) and class-switch recombination (CSR), resulting in the generation of antibodies with higher affinity and different isotypes (Mesin et al. 2016). In addition, the germinal center is also the site of the formation of memory B cells and antibody-secreting plasma cells (Suan et al. 2017). The formation and function of the germinal center (GC) are regulated by a variety of signals and factors. Among them, the activation and mediation of Tfh cells, which promote the formation and maintenance of the germinal center by releasing cytokines such as IL-21 and IL-4, are crucial (Crotty 2014). Antigenic stimulation is another key factor that triggers the initiation of the germinal center response by binding to immunoglobulins of B cells (Finney et al. 2018). Meanwhile, co-stimulatory molecules such as CD40 and CD40L activate and regulate the proliferation and differentiation of B cells through intercellular signaling pathways (Crotty 2014). On the other hand, some signaling inhibitors, such as PD-1 and PD-L1, play a negative regulatory role in the response of germinal centers, limiting their overactivation and damage (Mintz and Cyster 2020).

**Fig. 1:**
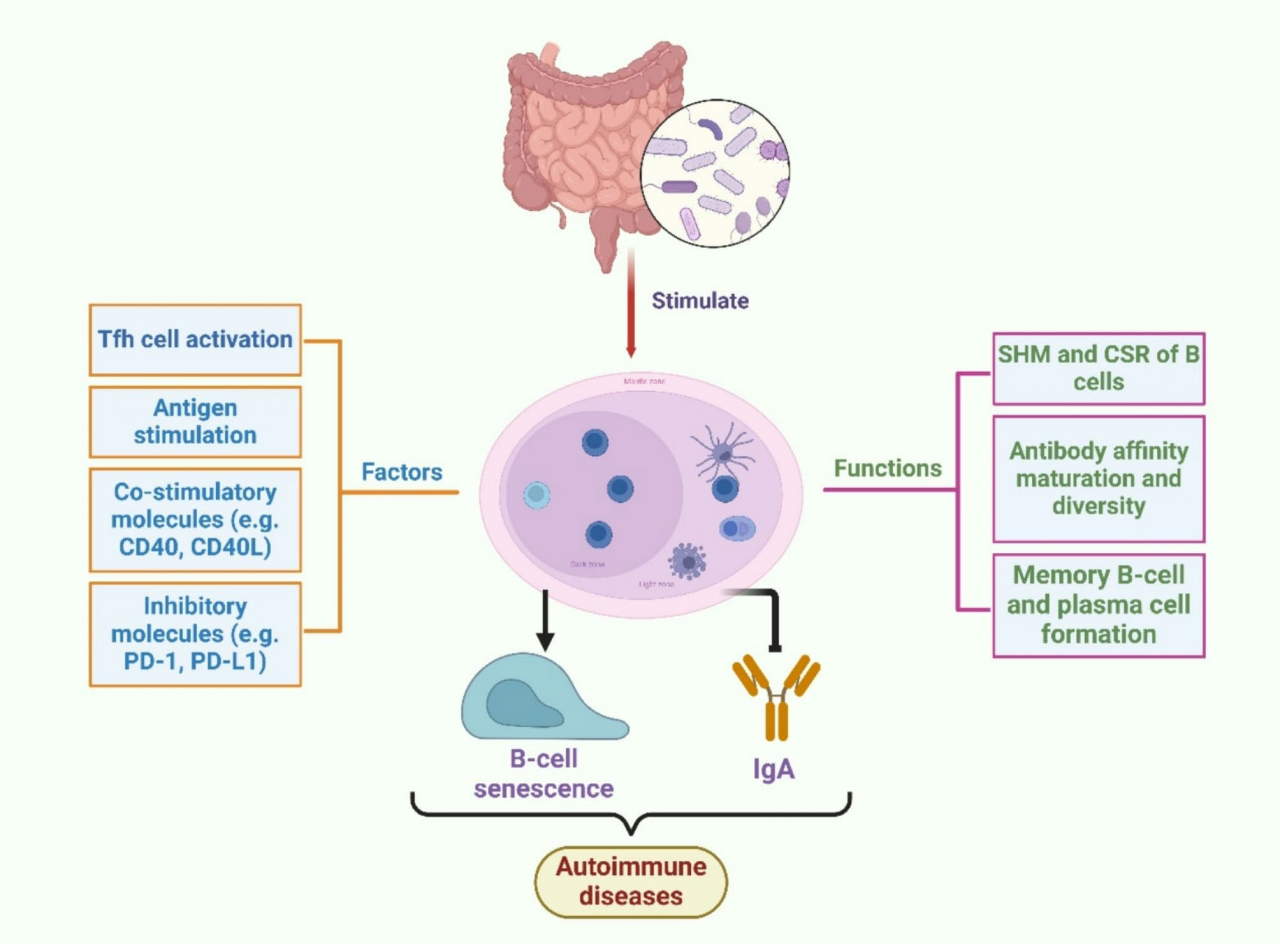
Gut microbiota stimulation induces alterations in germinal centers affecting the development of autoimmune diseases

Recently, it has been shown that sustained gut microbial stimulation can lead to cellular senescence of GC B cells, which may be associated with decreased production and diversity of intestinal immunoglobulin A (IgA), an alteration that induces dysbiosis of the gut microbiota (Kawamoto et al. 2023; Kawamoto and Hara 2024). In addition, mice with low parasite loads exhibit increased GC B cell counts and specific antibody titers, suggesting that differences in the composition of the gut microbiota may influence malaria severity by altering GC B cell counts and antibody responses (Waide et al. 2020). Importantly, the effect of gut microbiota on GCs has been linked to the course of autoimmune diseases (Rosser et al. 2020). NLRP12, a protein that regulates inflammation and plays a dual role in autoimmunity, is significantly altered in Nlrp12-/- B6/ lpr mice, especially in males. This alteration may lead to inhibition of terminal B-cell differentiation, restricted GC responses, reduced autoantibody production, and the expansion of potentially pathogenic T cells, suggesting that NLRP12 plays an important role in the regulation of B and T cell function and GC responses (Abdelhamid et al. 2023). CX 3 CR1 is a chemokine receptor, which has been shown in most previous studies to be positively associated with lupus disease progression with elevation of the ligand CX 3 CL1 (Nakatani et al. 2010; Inoue et al. 2005). However, a new study found that Cx3cr1 -/- MRL/lpr mice exhibited worsening glomerulonephritis and that low levels of CX 3 CR1 may have a protective effect, which is associated with abnormalities in the gut microbiota. In addition, Cx3cr1 -/- MRL/lpr mice activated monocytes to express ICOS-L after high-fat dietary treatment, which interacted with Tfh-expressed ICOS, which in turn facilitated a germinal center response, production of more autoantibodies, and the appearance of more atherosclerotic plaques (Cabana-Puig et al. 2023). Interestingly, in B6.Sle1b mice, STAT1 tyrosine 701 phosphorylation (STAT1-pS727) promoted germinal center (GC) responses, which drove the development of systemic lupus erythematosus (SLE), whereas STAT1-pS727 was not required for the response of GCs, Tfh cells, and antibodies to foreign antigens, suggesting that gut microbes may influence the development of autoimmune responses through different mechanisms to influence the onset and development of autoimmune responses (Chodisetti et al. 2020). In a study on arthritic mice, supplementation with gut microbe-derived butyrate was found to inhibit germinal center (GC) B cell and plasmablast differentiation and suppress arthritis in a Breg-dependent manner (Rosser et al. 2020). Segmented filamentous bacteria (SFB) in K/BxN mice can induce an increase in Tfh cells accompanied by a robust germinal center B cell response, which in turn promotes the development of autoimmune arthritis (Bates et al. 2021). Follicular regulatory T (Tfr) cells are important in regulating follicular helper T (Tfh) cell production and antibody production in the GC response, and SFB-induced arthritis correlates with a decrease in the Tfr cell-regulating molecule, CTLA-4, whose expression is correlated with the intensity of the T cell receptor (TCR) Nur77 signaling (Bates et al. 2021). Meanwhile, dysregulation of gut microbiota may also lead to alterations in Tfr cell number and function, and changes in Tfr cell number and function may lead to dysregulation of the GC response and production of abnormal autoantibodies (Fig. 1, by BioRender) (Ding et al. 2019). Therefore, restoring the balance of Tfr/Tfh cells by regulating the gut microbiota may be an effective way to treat RA.

## Interactions between gut microbiota and B cells: treatment of autoimmune diseases by gut microbiota-B cell pathway

B lymphocytes in gut-associated lymphoid tissue (GALT) have been implicated in the pathogenesis of autoimmune diseases, which also suggests new treatment strategies. By directly affecting the gut microbiota and/or regulating the metabolites derived from gut microorganisms, B cells play a key role, thereby inhibiting and interfering with autoimmunity.

At present, probiotic therapy has been evaluated in the treatment of a variety of diseases (Cheng et al. 2021; Li et al. 2021). Probiotics can regulate immune responses within the host mucosa and the balance of flora in the intestine (Hrdý et al. 2020), and these potential interactions may also be relevant for the treatment of autoimmune diseases. Commensals under current study as therapeutic probiotics primarily include Lactobacillus, Yeast, Probiotic Bacillus, Bifidobacterium, Clostridium, and others (Gareau et al. 2010).

The potential of probiotics in the treatment of autoimmune diseases has been explored, particularly in relation to imbalanced microbiomes, with the promise of treating autoimmune diseases through probiotics interacting with the gut microbiota and modulating host immune responses (Mousa et al. 2023). In terms of mechanisms that have been investigated, probiotics can induce an increase in the number of IgA cells that secrete more IgA into the gut lumen to maintain homeostasis within the intestinal mucosa, which may result in the production of some anti-inflammatory cytokines, as well as increase the levels of IL-10 and TGF-β (Di Giacinto et al. 2005). In addition, oral administration of probiotics may increase Breg cells, thereby limiting the inflammatory response (Liang et al. 2023).

These results suggest that probiotics can inhibit autoimmune-induced inflammation to some extent and modulate the body’s immune response at the B-cell level. Although probiotics show great potential in the treatment of disease, more in-depth studies are needed to determine their effectiveness and the optimum approach for their use.

Fecal microbiota transplantation (FMT) is a treatment that transplants the functional microbiota in the feces of healthy donors into the gastrointestinal tract of the patient to rebuild a new gut microbiota balance, thereby restoring the normal microbial composition in the intestinal tract. It is a widely used treatment for refractory Clostroides difficile infection (Matsuoka et al. 2014; Waller et al. 2022).

There have been many studies on the pathogenesis and treatment of autoimmune diseases through FMT, and this therapy is considered to have great potential (Marietta et al. 2020; Belvoncikova et al. 2022). Multiple autoimmune diseases such as Multiple sclerosis, Systemic lupus erythematosus, Rheumatoid arthritis, Psoriatic arthritis, Sjogren’s disease, Type 1 diabetes, and Idiopathic thrombocytopenic purpura have been studied with FMT (Belvoncikova et al. 2022). Recent an EXPLORER trial has shown that FMT has a positive effect on controlling SLE disease activity (Huang et al. 2022). FMT treatment leads to potentially beneficial changes in B-cell subpopulations and gene expression, suggesting that it may be a promising approach for the treatment of SLE, particularly in the context of targeted therapies against in the cause of lupus. (Zheng et al. 2023).

In autoimmune diseases, the functional activity of B cells is usually disturbed to varying degrees. However, by modifying the composition of the intestinal microbiota, in particular, through fecal microbial transplantation (FMT), it is expected that the normal function of B cells in the organism can be effectively restored. This strategy potentially contributes to modulating the development of autoimmune diseases. Although this concept offers a compelling direction for the future treatment of autoimmune diseases, more in-depth studies are needed to fully validate the safety and efficacy of this therapy.

## Discussion and further directions

**Fig. 2:**
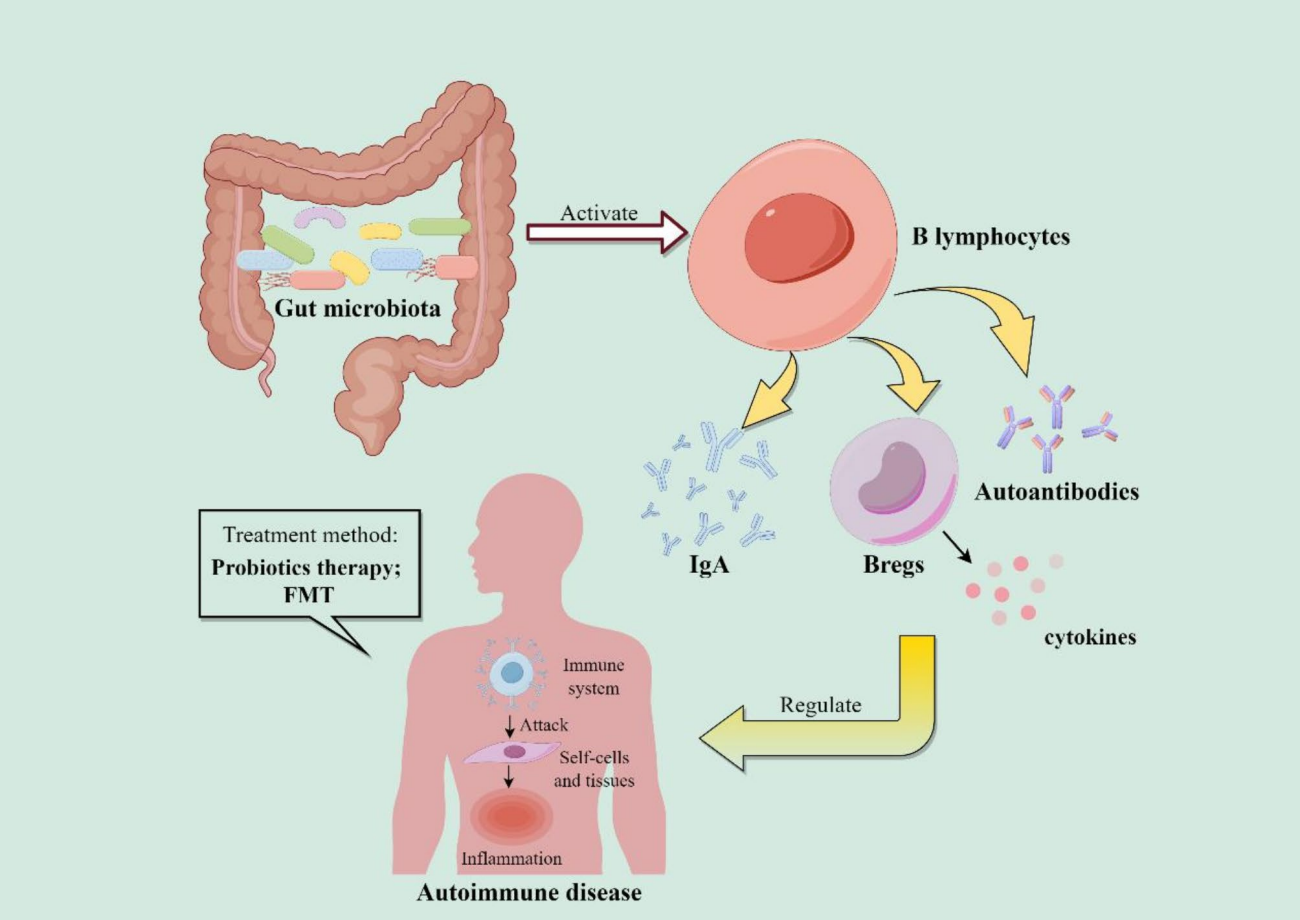
The effect of gut microbiota on B lymphocytes mediates the development of autoimmune diseases

At present, with the development and progress of science and technology, such as high-throughput sequencing (Aron-Wisnewsky and Clément 2016; Ibrahim et al. 2022), the study of gut microbiota has been more in-depth, and we have a new understanding of autoimmune diseases and some treatment strategies. However, the pathogenesis of autoimmune diseases is still the subject of a variety of hypotheses. The aim of this paper is to explore the interplay between the gut microbiota and the human immune system, with a special focus on the key role of B lymphocytes in regulating gut microbiota and autoimmune diseases. Studies have shown a correlation between alterations in the gut microbiota and the development of autoimmune diseases, but the specific role of B cells in this relationship has not been fully investigated. This paper summarizes the impact of the gut microbiota - B cell pathway on autoimmune diseases and how these mechanisms may serve as potential therapeutic targets (Fig. 2, by Figdraw), providing organic insights into the field.

The gut microbiota also affects other immune system cells, such as antigen-presenting cells (e.g., dendritic cells and macrophages), in several ways. On the one hand, the gut microbiota can influence the activation status of antigen-presenting cells, and it has been found that gut microbiota metabolites may affect the maturation and activation of antigen-presenting cells, e.g., butyrate and propionate can act to inhibit the maturation of DCs through the transport protein (Slc5a8)-dependent histone deacetylase (Singh et al. 2010), and butyrate enhances the antimicrobial function of macrophages (Schulthess et al. 2019). On the other hand, gut microbiota can also influence cytokine production in antigen-presenting cells, e.g., short-chain fatty acid butyrate, a metabolite derived from gut microbiota, can act on DCs and macrophages to modulate cytokine/chemokine production (Nastasi et al. 2015; Chang et al. 2014). In autoimmune diseases, the gut microbiota can influence disease progression by affecting the function of antigen-presenting cells, and it has been found that secondary bile acids modulate dendritic cell function via the TGR5-cAMP-PKA pathway to reduce the severity of experimental autoimmune uveitis (EAU) in mice and that this alteration correlates with the composition of the gut microbiota in EAU mice (Hu et al. 2021). In addition, P. distasonis-derived metabolites isoLCA and 3-oxoLCA were found to promote macrophage M2 polarization, which significantly ameliorated the inflammatory state in arthritic mice (Sun et al. 2023). Fusobacterium nucleatum causes worsening of arthritis in a mouse model of CIA through the release of FadA-containing outer membrane vesicles, in which FadA triggers synovial inflammation by activating the Rab5a-YB-1 pathway in synovial macrophages (Hong et al. 2023). These findings highlight the important role of the gut microbiota in immune regulation and provide new perspectives and potential targets for studying the pathogenesis of autoimmune diseases.

A large number of studies have been conducted on the effects of the gut microbiota on T cells. To summarize, the gut microbiota affects T cell function and activity through multiple pathways. First, they regulate T cell differentiation and promote or inhibit the development of specific subpopulations, such as Th1, Th2, Th17, and Treg (Kim 2022). Secondly, the gut microbiota is involved in regulating inflammatory responses and influencing T-cell activity. In addition, they produce signaling molecules, such as bacterial carboxylic acids and polysaccharides, which indirectly affect B cell activity and antibody production by influencing T cell function (Shim et al. 2023; Ivanov et al. 2022). Finally, the gut microbiota also modulates immune tolerance, e.g., by increasing the number of Treg cells, which in turn affects the overall immune response and B cell function (Brown et al. 2019). Together, these pathways constitute a combined effect of the gut microbiota on T and B cells, directly or indirectly influencing the onset and progression of autoimmune diseases.

In addition to the intestinal tract, the oral cavity, urogenital tract, and other sites also house rich microbial colonies (Aggarwal et al. 2023), and their effects on B cells and autoimmune diseases are worthy of our attention and research. Therefore, we need to understand in more detail how the microbiota affects the function of B cells and how B cells regulate immune responses through different mechanisms, which will help to reveal the pathogenesis of autoimmune diseases.

By intervening in the gut microbiota - B cell pathway, we can develop new therapeutic strategies for the control of autoimmune diseases. Different autoimmune diseases may have different gut microbiota and B cell characteristics. Therefore, future treatments may be more individualized, with treatment plans based on each patient’s microbiota and immune characteristics.

## Conclusion

Findings in recent reports highlight the important roles of the gut microbiota-B cell pathway in autoimmune diseases. These complex interactions between gut microbiota and B cells during autoimmune pathogenesis involve multiple aspects of biology, including inflammatory responses, immune regulation and metabolic homeostasis, and potentially provide opportunities for the treatment of autoimmune diseases through the development of drugs with relevant targets. The studies herein provide new perspectives to better understand the occurrence and development of autoimmune diseases. These findings lay the foundation for the development of more effective treatments and individualized therapeutic regimens, and future studies will further reveal the mechanisms of these complex relationships and provide more innovative solutions for the treatment of autoimmune diseases.

## Data Availability

Not applicable.

## Abbreviations

Bregs: Regulatory B cells
GALT: Gut-associated lymphoid tissue
SLE: Systemic lupus erythematosus
RA: Rheumatoid arthritis
IBD: Inflammatory bowel disease
AIH: Autoimmune hepatitis
T1D: Type 1 diabetes
FMT: Fecal microbiota transplantation
SCFAs: Short-chain fatty acids
IgA: Immunoglobulin A
IgM: Immunoglobulin M
IgG: Immunoglobulin G
IL-10: Interleukin-10
TNF-α: Tumor necrosis factor-alpha
IFN-γ: Interferon gamma
NK: Natural killer cells
PP: Peyer’s patches
LPL: Lamina propria lymphocytes
TGF-β: Transforming growth factor-beta
Tfh: T follicular helper cells
Tfr: T follicular regulatory cells
GC: Germinal center
APCs: Antigen-presenting cells
ITAM: Immunoreceptor tyrosine-based activation motif
STAT1: Signal transducer and activator of transcription 1
AhR: Aryl hydrocarbon receptor
ICOS: Inducible T-cell costimulator
CX3CR1: C-X3-C motif chemokine receptor 1
EBV: Epstein-barr virus
LMP1: Latent membrane protein 1
Tregs: Regulatory T cells
HUMSC: Human umbilical cord mesenchymal stem cells
SPF: Specific pathogen free
GF: Germ-free
ICOS-L: Inducible T-cell costimulator ligand
HLA-B27: Human leukocyte antigen B27
MBP: Myelin basic protein
